# Middle meningeal artery embolization: an emerging treatment for non-acute subdural hematomas

**DOI:** 10.31744/einstein_journal/2026CE2043

**Published:** 2025-12-11

**Authors:** Thiago Gebrin, Thiago Giansante Abud, Andre Felix Gentil, Arthur Werner Poetscher

**Affiliations:** 1 Unidade de Pronto Atendimento Hospital Municipal Gilson de Cássia Marques de Carvalho Hospital Israelita Albert Einstein São Paulo SP Brazil Unidade de Pronto Atendimento (UPA), Hospital Municipal Gilson de Cássia Marques de Carvalho; Hospital Israelita Albert Einstein, São Paulo, SP, Brazil.; 2 Hospital Israelita Albert Einstein São Paulo SP Brazil Hospital Israelita Albert Einstein, São Paulo, SP, Brazil.

Dear Editor,

Subdural hematoma (SDH) is a type of intracranial hemorrhage that is characterized by the accumulation of blood in the subdural space.^( 1 )^ Although numerous conditions, such as coagulopathy, neoplasms, vascular malformations, aneurysms, and alcohol and cocaine use can lead to SDH, acute SDH is more often caused by head trauma.^( 1 )^ An acute SDH may resolve by reabsorption or, alternatively, it can progress to chronic SDH, generally by encapsulation and a complex process involving inflammation, angiogenesis, and fibrinolysis.^( 2 , 3 )^ The incidence of chronic SDH is reportedly increasing, probably due to population aging and increased use of antiplatelet and anticoagulant medication.^( 2 )^

The standard treatment for thick, compressive, or symptomatic SDH remains neurosurgical evacuation.^( 4 )^ However, middle meningeal artery embolization (MMAE), a neurointerventional endovascular procedure that occludes the blood vessels contributing to the growth of chronic SDH,^( 1 )^ has recently gained attention as a promising technique for managing this condition. In November 2024, three notable randomized controlled trials (RCTs)^( 5 - 7 )^ that evaluated MMAE were published.

The EMBOLISE^( 5 )^ trial was a multicenter, open-label, adaptive-design RCT conducted in the United States.^( 5 )^ It included 400 patients with symptomatic, thick, or midline-shifting subacute or chronic SDH with an indication for surgery (burr-hole or craniotomy).^( 5 )^ Patients were randomized to MMAE plus surgery (197 patients) or surgery alone (203 patients) groups, utilizing the Onyx liquid embolic system (Medtronic, study sponsor and collaborator) as the embolic agent.^( 5 )^ A statistically significant 64% relative reduction in hematoma recurrence or progression requiring repeat surgery within 90 days was observed.^( 5 )^ No significant inter-group difference was found in neurological deterioration at 90 days (measured using the modified Rankin Scale) in a non-inferiority analysis.^( 5 )^ Mortality was slightly higher in the MMAE group (5.1% *versus* 3.0%), but was not considered to be related to the procedure or embolic agent.^( 5 )^ No significant differences were observed in the incidence of stroke, although two patients in the MMAE group experienced procedure-related disabling strokes within the first month.^( 5 )^ Importantly, the confidence intervals for additional efficacy endpoints and safety outcomes were not adjusted for multiplicity and should not be used for statistical inference.^( 5 )^

The MAGIC-MT^( 6 )^ was a multicenter, open-label RCT conducted in China that enrolled 727 patients with symptomatic non-acute SDH with mass effect who were previously independent in everyday activities.^( 6 )^ Patients requiring craniotomy, emergency evacuation, or presenting with bilateral SDH with uncertain laterality of symptoms were excluded.^( 6 )^ Patients were first categorized according to the need for burr-hole drainage, as judged by the treating neurosurgeon.^( 6 )^ They were then randomized to MMAE (365 patients) or usual care (362 patients), which included burr-hole drainage, medical treatment, or both.^( 6 )^ Onyx was again used as the embolic agent, although the study sponsors (including Medtronic) reportedly had no significant involvement in the trial's conduct.^( 6 )^ The primary outcome, symptomatic SDH recurrence or progression of SDH within 90 days, showed no statistically significant difference between the two groups.^( 6 )^ Exploratory subgroup analyses suggested possible benefits for patients not undergoing burr-hole drainage, those with a midline shift <10mm, or those who had never smoked; however, these results were not adjusted for multiplicity and cannot support causal inference.^( 6 )^ Secondary outcomes included both clinical and imaging measures, but these were also not multiplicity-adjusted.^( 6 )^ Interestingly, unlike the EMBOLISE^( 5 )^ trial, a significant 42% relative reduction in the rate of serious adverse events (including death) within 90 days was observed in the MMAE group, although the 90-day mortality and rates of selected adverse events were unaffected.^( 6 )^ MMAE-related complications included one case of facial nerve palsy and two allergic reactions to the contrast agent.^( 6 )^ A notable limitation was the high proportion of patients who had previously undergone surgery, complicating the evaluation of the efficacy of MMAE in conservatively treated cases.^( 6 )^

The STEM^( 7 )^ trial was an international, multicenter, open-label RCT that evaluated 310 patients with symptomatic chronic SDH >10mm on imaging.^( 7 )^ Patients were previously submitted to standard surgical or non-surgical treatment, and those undergoing craniotomy or those with a previous modified Rankin scale score >1 were excluded.^( 7 )^ Both unilateral and bilateral SDH were eligible, provided the inclusion and exclusion criteria were met.^( 7 )^ Patients were randomized to MMAE plus standard therapy (surgical or non-surgical, 149 patients) or standard therapy alone (161 patients).^( 7 )^ Surgical standard treatment included either burr-hole evacuation or subdural evacuating port system drainage.^( 7 )^ Unlike the previous two trials,^( 5 , 6 )^ the Squid liquid embolic agent (Balt USA, study sponsor and collaborator) was used in this trial.^( 7 )^ The primary efficacy outcome, a composite of recurrent or residual SDH >10mm, reoperation, or major adverse events ( *e.g* ., disabling stroke, myocardial infarction, or death by neurological causes) within 180 days, showed a statistically significant 64% reduction in the odds ratio.^( 7 )^ The most frequent event in both groups was reoperation (20% combined).^( 7 )^ No significant difference in the primary safety outcome (composite of disabling stroke or death within 30 days) was found between the two groups (both groups: 3%).^( 7 )^ However, the 180-day mortality rate was slightly higher in the MMAE group (8% *versus* 5%), although the difference was not statistically significant.^( 7 )^ Again, the widths of some confidence intervals were not adjusted for multiplicity, preventing its use in place of hypothesis testing.^( 7 )^

A meta-analysis^( 8 )^ of these three RCTs^( 5 - 7 )^ revealed mixed findings. Using a random-effects model, which was deemed preferable due to between-study heterogeneity, MMAE was found to significantly reduce reoperation rates at 90 days by 57% in surgical patients, and progression or surgical rescue in non-surgical patients by 64%.^( 8 )^ However, there were no significant differences in the trial's pooled primary outcomes (mostly recurrence or progression) between the combined groups or functional outcomes.^( 8 )^ Notably, a fixed-effects model showed a significant protective effect of 50% overall, 40% in surgical patients, and 64% in those managed non-surgically.^( 8 )^

Recently, a prospective single-center RCT conducted in Brazil from 2021 to 2024 evaluated the effectiveness of MMAE using Histoacryl as an adjuvant to surgical treatment of chronic SDH.^( 9 )^ A total of 76 hematomas were randomized to receive either surgery alone or surgery followed by MMAE.^( 9 )^ Recurrence occurred in 4.2% of the MMAE group and 12.5% in the control group, with no significant differences in mortality, length of stay, or complication rates.^( 9 )^ Notably, embolization was performed using N-butyl-2-cyanoacrylate (Histoacryl) mixed with Lipiodol, which has demonstrated good distal penetration and safety.^( 9 )^ Although the difference in recurrence rates was not statistically significant, this study supported MMAE with Histoacryl as a promising adjunctive approach for the management of chronic SDH, especially in resource-limited settings where alternative agents, such as Onyx or Squid, are cost-prohibitive.^( 9 )^

In summary, MMAE has emerged as a promising adjunct or potential alternative to conventional neurosurgical approaches for the management of non-acute SDH. As of November 2024, 21 trial protocols on this topic have been registered, and many of these trials were ongoing and recruiting.^( 8 )^ The publication of further RCTs will likely prompt updated meta-analyses, ideally incorporating advanced methods such as trial sequential analysis^( 10 )^ to better clarify the true efficacy, safety, cost-effectiveness, and optimal embolic agent for this increasingly relevant intervention.^( 8 )^

## DATA AVAILABILITY

The underlying content is contained within the manuscript.
